# Phenotype and Treatment of Breast Cancer in HIV-Positive and -Negative Women in Cape Town, South Africa

**DOI:** 10.1200/JGO.2015.002451

**Published:** 2016-03-30

**Authors:** Lizanne Langenhoven, Pieter Barnardt, Alfred I. Neugut, Judith S. Jacobson

**Affiliations:** **Lizanne Langenhoven** and **Pieter Barnardt**, Stellenbosch University, South Africa; **Alfred I. Neugut** and **Judith S. Jacobson**, Mailman School of Public Health and Herbert Irving Comprehensive Cancer Center, Columbia University; and **Alfred I. Neugut**, College of Physicians and Surgeons, Columbia University, New York, NY.

## Abstract

**Purpose:**

An estimated 5.9 million people in South Africa are infected with HIV. Because antiretroviral therapy has made infection with HIV a treatable, chronic condition, HIV-infected individuals are now surviving to middle and older age. We investigated the implications of HIV status for breast cancer in South Africa.

**Methods:**

We compared clinical and demographic characteristics of women newly diagnosed with a first primary breast cancer at Tygerberg Hospital, Cape Town, South Africa, from January 2010 to December 2011 by HIV status. We then compared HIV-positive patients with HIV-negative controls, matched 2:1 on age and ethnicity, with respect to chemotherapy regimens, toxicities, completion of systemic chemotherapy, and changes in CD4 cell count.

**Results:**

Of 586 women with breast cancer, 31 (5.3%) were HIV positive, 420 (71.7%) were HIV negative, and 135 (23%) were untested for HIV. Women with HIV were younger than other women (*P* < .001). The groups did not differ in regard to stage at presentation, histologic subtype, tumor grade, nodal involvement, or hormone receptor positivity. More than 84% of patients who initiated systemic chemotherapy, regardless of HIV status, completed it without serious toxicity. Among HIV-positive patients receiving chemotherapy, the mean baseline CD4 cell count was 477 cells/µL (standard deviation, 160 cells/µL), and the mean nadir was 333 cells/µL (standard deviation, 166 cells/µL).

**Conclusion:**

HIV-infected women were younger at breast cancer diagnosis than HIV-negative women but otherwise similar in phenotype and completion of chemotherapy. Longer term follow-up is needed to evaluate the effects of HIV, antiretroviral therapy, and chemotherapy on the survival and quality of life of patients with breast cancer.

## INTRODUCTION

Of the 35.3 million people worldwide who were estimated to be infected with HIV/AIDS in 2012, 25 million (71%) lived in Sub-Saharan Africa, including 6.1 million in South Africa, the country with the largest number of people living with HIV/AIDS in the world.^1^

In the United Kingdom, between 1996 and 2008, the life expectancy of HIV/AIDS-infected people increased by an average of 15 years, presumably because of antiretroviral therapy (ART). By the end of the period, the life expectancy of people with HIV/AIDS was only 13 years shorter than that of the general population of the United Kingdom.^2^ In South Africa, although no comparable data are yet available, it is clear that persons with HIV infection are living longer as a result of ART than infected people did before the use of ART. Therefore, like uninfected people, those with HIV infections will incur increasing risks for the epithelial malignancies associated with aging.

By 2010, 19% of all deaths in the Swiss HIV Cohort Study were attributable to non–AIDS-defining cancers.^3^ Persons with HIV had a higher than average risk for anal cancer, lung cancer, certain head and neck cancers, hepatocellular carcinoma, and Hodgkin lymphoma, but not for breast cancer, prostate cancer, or colorectal cancer. Little is known about the phenotype of these malignancies and about the outcomes of standard oncologic treatment among HIV-positive patients, including those receiving ART.^4-9^

In a series of 1,092 black women consecutively diagnosed with breast cancer in Soweto, South Africa, between January 2006 and July 2012,^10^ 19.7% were found to be HIV positive. Nearly a quarter of the HIV-positive women were diagnosed with a CD4 count less than 200 cells/µL. The HIV-positive women were younger at diagnosis than those who were HIV negative or not tested, but they did not differ in tumor characteristics or prognostic factors. This finding was in stark contrast to anecdotal reports of a more aggressive phenotype in HIV-positive women.

It was recently shown that HIV-positive patients were diagnosed with more advanced-stage cervical cancer than their HIV-negative peers, had a decreased likelihood of completing radical chemoradiotherapy, and fared worse overall in regard to treatment-related toxicity, especially when chemotherapy was used in conjunction with radiotherapy.^11,12^ Whether these findings also apply to women diagnosed with breast cancer remains to be determined because the risk factors associated with these epithelial malignancies are inherently different.

Given that breast cancer is the most common non–AIDS-defining cancer in the global female population^13^ and that risk increases with age, the proportion of patients with breast cancer in South Africa who are HIV positive is likely to increase in the next few years. Yet at present, no specific guidelines are available to clinicians caring for patients with both diagnoses. A case series in the era before widespread use of ART found that such patients had poor tolerance for systemic cytotoxic therapy and that most experienced progressive immune impairment with progression of HIV. The authors cautioned that hormonal therapy, rather than systemic chemotherapy, should be considered in the adjuvant setting.^14^ The primary objective of this study is to compare HIV-positive and -negative women with breast cancer in terms of their clinical and demographic characteristics and tolerance of standard chemotherapy regimens.

## METHODS

After obtaining approval from our Ethical Review Board, we reviewed the charts of all women registered with a diagnosis of breast cancer on the oncology service at the Tygerberg Academic Hospital in Cape Town, South Africa, from January 2010 through December 2011. Of 816 patients whose records we reviewed, we excluded 154 for insufficient data, 47 for a prior diagnosis of breast cancer, 12 for male sex, 16 for ductal carcinoma in situ, and five for benign breast disease.

We then compared patients by HIV status with respect to age at diagnosis, ethnic group, and menopausal status. We also compared them with respect to disease phenotype (stage at diagnosis,^15^ histologic subtype, grade of differentiation, nodal status, and positivity to estrogen receptor, progesterone receptor [PR], and human epidermal growth factor receptor 2). Chemotherapy tolerance and toxicities were captured for the HIV-positive subgroup and an HIV-negative comparison group matched 2:1 on age and ethnicity. We determined how many of the assigned chemotherapy cycles were completed. Allowance was made for interruptions or postponements as well as dose reductions as a result of toxicities. Patients were considered to have completed chemotherapy if they had completed the number of cycles assigned to them at the beginning of treatment, regardless of dose reduction or treatment interruption. Toxicity was scored according to the National Cancer Institute Common Terminology Criteria for Adverse Events (version 4.0).^16^ We assigned a toxicity grade to each patient at each cycle for the following hematologic parameters: hemoglobin, WBC count, platelets, neutrophils, lymphocytes, and CD4 count. Clinical toxicities evaluated were nausea and vomiting, alopecia, thrombophlebitis, and weight loss. The CD4 nadir was defined as the lowest value recorded at any time after initiation of treatment. It is standard practice to measure the CD4 cell count at cycle 3 and on completion of chemotherapy.

Statistical analysis was performed using Statistica software (Dell Statistica, Tulsa, OK). Where data were incomplete, an unknown category was created before data analysis. The statistical significance of differences among HIV status groups was evaluated by χ^2^ test and analysis of variance, with significance set at *P* < .05.

## RESULTS

Of the 586 patients who met the inclusion criteria, 451 (77%) were tested for HIV and 135 (23%) were not; 31 patients (5.3%) tested positive and 420 (71.7%) tested negative (Table 1). A total of 359 patients (61.3%) were of mixed race, 158 (26.9%) were of European descent, and 69 (11.8%) were of African descent.

**Table 1:**
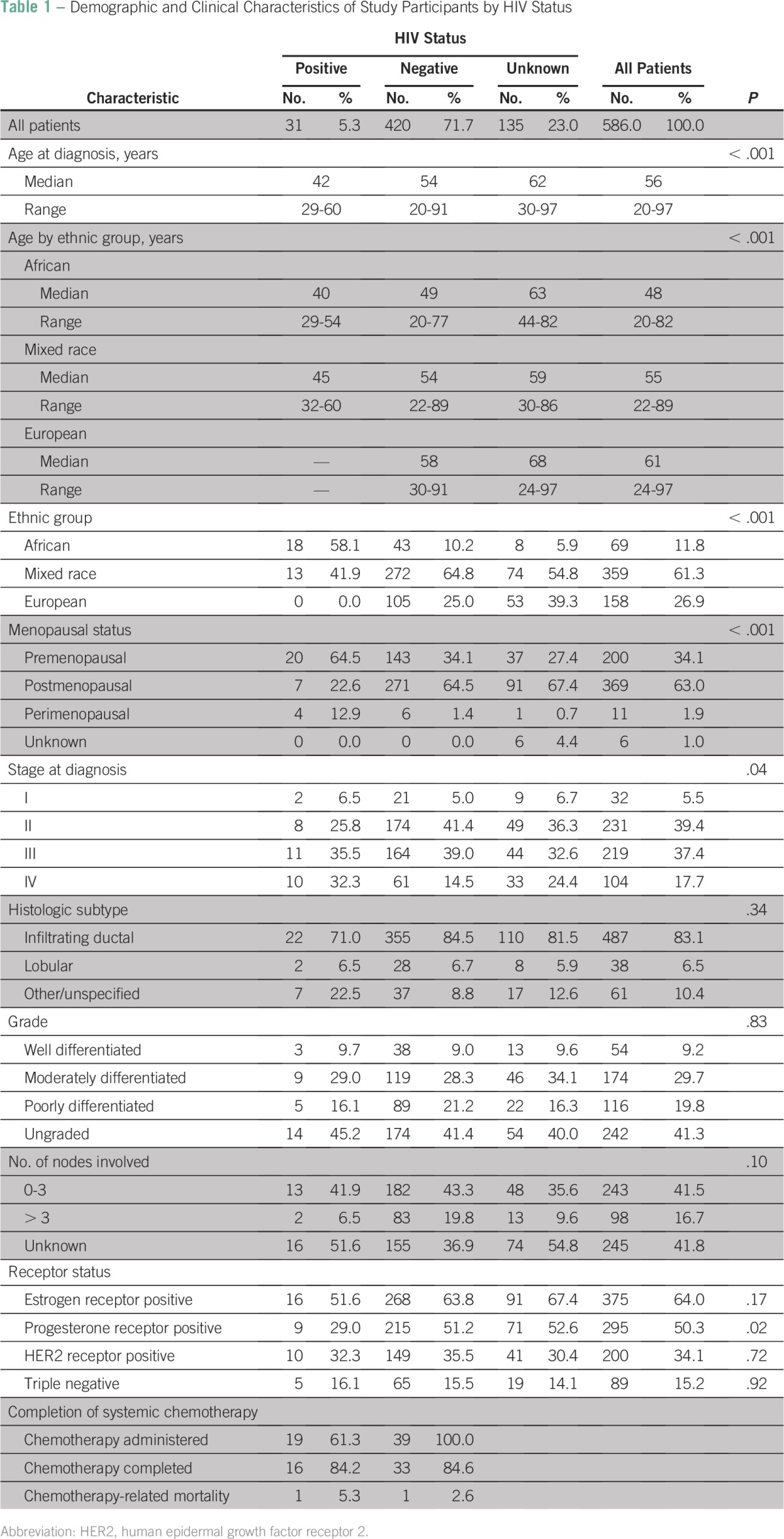
Demographic and Clinical Characteristics of Study Participants by HIV Status

Ethnicity was strongly associated with HIV status; 27.1% of African patients but only 3.6% of those of mixed race and none of those of European background were HIV positive (*P* < .001). Nearly 90% of African patients were tested for HIV; mixed-race patients and those of European descent were much less likely to be tested. The patients’ median age at presentation was 56 years (range, 20 to 97 years). The median age of the HIV-positive group was 42 years, that of the HIV-negative group was 54 years, and that of the group with unknown HIV status was 62 years (*P* < .001). Within each ethnic group, HIV-positive patients were younger than HIV-negative patients, who were younger than untested patients (*P* < .001). Within the HIV-positive and -negative groups, African patients were younger than patients of mixed race and patients of European background. In the untested group, the African patients were older than those of mixed race.

Fifty-five percent of all patients were diagnosed with stage III or IV breast cancer, including 68.8% of HIV-positive patients, 54% of HIV-negative patients, and 57% of untested patients (*P* < .001 for the association of HIV status with stage). Overall, 488 patients (83.1%) had infiltrating ductal carcinoma; only 38 patients (6.5%) had infiltrating lobular carcinoma. The 61 remaining patients had tumors of other rare or unspecified histologic subtypes. Although HIV-positive patients were more likely than others to have rare epithelial subtypes, the differences were not statistically significant. The grade of tumor differentiation was not associated with HIV status.

Of the 341 patients offered axillary sampling (because they had surgical treatment and were younger than 70 years), 243 (71.3%) had fewer than four nodes involved. Nodal status was not associated with HIV status.

With regard to hormonal prognostic markers, HIV-negative and untested patients were nearly twice as likely as HIV-positive patients to have PR-positive disease (*P* = .02). The groups did not differ with respect to estrogen receptor, human epidermal growth factor receptor 2, or triple-negative status (Table 1).

Of the 31 HIV-positive patients, 22 (71%) were known to be HIV positive at breast cancer diagnosis; the other nine were diagnosed at their first clinic visit. Twelve patients had been on ART for 3 months or longer. All patients newly diagnosed with HIV were referred for initiation of ART. Two HIV-positive patients had pulmonary tuberculosis at the time of their cancer diagnosis, one patient was newly diagnosed with a pulmonary aspergilloma, and one patient was diagnosed with *Staphylococcus* pneumonia.

Of the 31 HIV-positive patients, five failed to return to the hospital for surgical or other treatment after staging examinations were performed. Another five patients did not receive chemotherapy because of comorbidities (eg, ischemic heart disease). One patient presented with stage I breast cancer and was managed with surgery only. One patient had just begun chemotherapy and had no record of toxicity at time of data analysis. Therefore, we report on toxicities among 19 HIV-positive patients who received systemic chemotherapy and 39 matched HIV-negative controls.

In the HIV-positive group, 16 patients received an anthracycline-based regimen in combination with cyclophosphamide and/or fluorouracil. The other three patients received a cyclophosphamide/methotrexate/fluorouracil (CMF) regimen because of cardiac compromise.

Three HIV-positive patients did not complete their assigned chemotherapy. The first patient discontinued in the middle of chemotherapy but returned 7 months later with local progression of her breast cancer. The second patient completed four cycles of palliative chemotherapy for stage IV disease before discontinuing treatment. She was eventually ascertained to have died, possibly as a result of chemotherapy-related toxicity. The third patient completed four cycles of chemotherapy but discontinued treatment after her tumor spread to the contralateral breast.

In the HIV-negative group, 35 patients received an anthracycline-based regimen in combination with cyclophosphamide and/or flourouracil, and four patients received CMF. Six patients in the HIV-negative cohort did not complete assigned chemotherapy. Two patients developed disease progression on treatment; three patients were lost to follow-up or transferred to a different hospital; and one patient died after three cycles of CMF, possibly as a result of treatment-related toxicity. Thus, 84% of both HIV-positive and HIV-negative patients completed their assigned chemotherapy (Table 1).

Table 2 compares the toxicity parameters of the HIV-positive and -negative subgroups. Alopecia, thrombophlebitis, and nausea and vomiting were the most frequently observed clinical toxicities in both groups (data not shown). No statistically significant difference between the HIV-positive and -negative subgroups could be demonstrated for hemoglobin, WBC count, platelets, and neutrophils (Table 2). However, HIV-positive patients had more lymphopenia than HIV-negative patients; five HIV-positive patients (26.4%) compared with no HIV-negative patients developed grade 3 or 4 toxicity (*P* = .001).

**Table 2:**
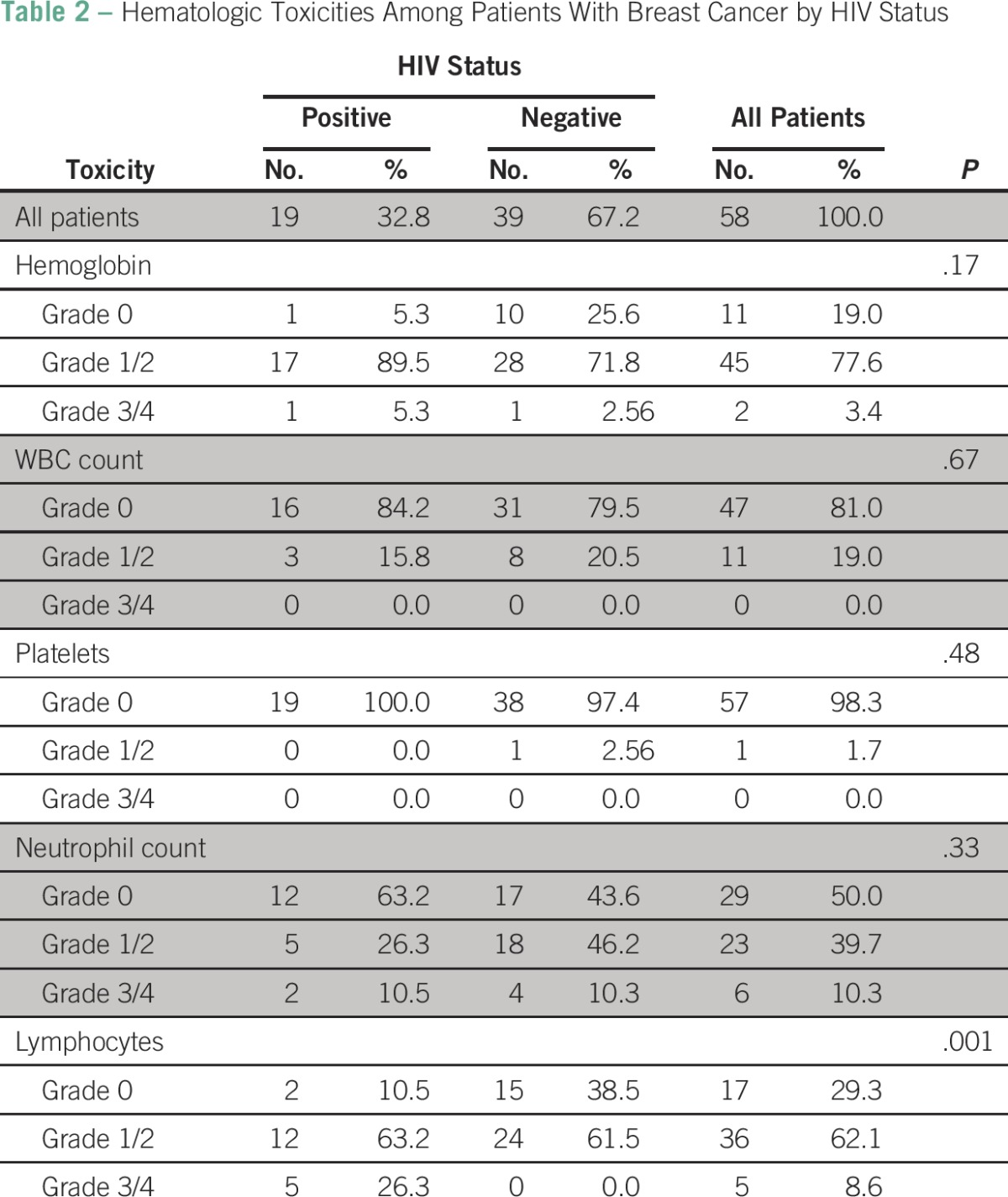
Hematologic Toxicities Among Patients With Breast Cancer by HIV Status

**Fig 1:**
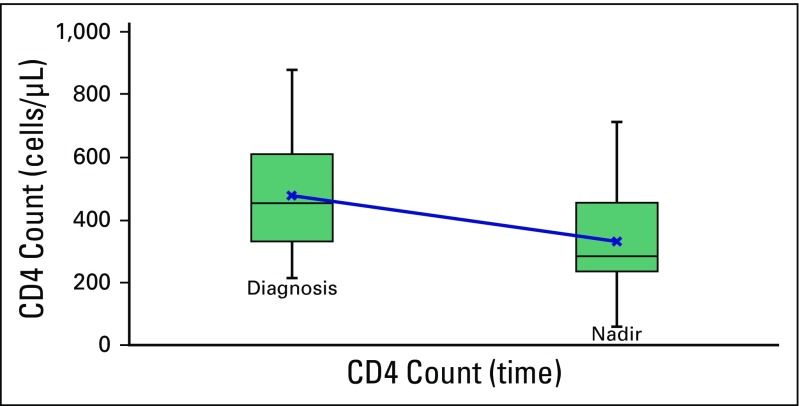
Trend of CD4 count during chemotherapy treatment.

Among patients in the HIV-positive cohort, the mean CD4 cell count at diagnosis was 402 cells/µL (standard deviation [SD], 244 cells/µL; range, 66 to 1,075 cells/µL). Among the 15 patients who received chemotherapy and had complete serial CD4 values, the mean CD4 cell count at diagnosis was 477 cells/µL (SD, 160 cells/µL), and the mean nadir was 333 cells/µL (SD, 166 cells/µL; *P* < .001; Fig 1). Five patients whose CD4 nadir was not recorded were excluded from the nadir value analysis.

Although it did not affect the completion of their planned chemotherapy, two patients required an adjustment to their ART regimen during chemotherapy, one as a result of a grade 4 anemia and the other as a result of a deterioration of her glomerular filtration rate.

## DISCUSSION

In our cohort, the majority of HIV-positive patients (58.1%) were of African descent, and the HIV-positive group was much younger than the HIV-negative group (42 *v* 54 years, respectively). Two important arguments in the literature are relevant to these observations. First, African American women are known to be younger at breast cancer diagnosis than American women of European descent. Second, Voutsadakis and Silverman^17^ argued that HIV-infected women with breast cancer are younger than other women with breast cancer because HIV-infected women are younger than HIV-negative women in the general population. Supporting that argument, in South Africa, the highest prevalence of HIV has been recorded in African women of reproductive age.^18,19^ The association of menstrual status with HIV status reflects the relative youth of the HIV-positive patients, most of whom were well below the mean age at menopause in the general population, which is currently estimated to be 51.4 years.^20^

The HIV prevalence of 5.3% observed in the total sample of 586 patients was less than the prevalence of 16% reported for the general population of South Africa. However, our institution is located in the Western Cape province, where HIV prevalence is known to be lower than elsewhere in the country.

Because HIV remains highly stigmatized in South Africa, obtaining accurate prevalence data for the general population is challenging. Current HIV infection estimates are based on indirect measures, such as antenatal incidence data, shifts in mortality patterns, and the analysis of annual surveys.^18,19^ The prevalence of HIV among patients in our sample who were tested was 7.1%, not much lower than the reported national prevalence of 7.7% in women age 55 to 59 years. However, 135 of our patients (23%) were not tested. The dilutional effect of including the untested, predominantly older patients with breast cancer in the prevalence calculation became evident when we examined the data by age group. Among the African patients, 26.1% tested positive for HIV; their median age was 40 years (range, 39 to 45 years). The prevalence of HIV among patients of mixed race and those of European background was less than the expected national average but still consistent with the age/race distribution in other samples.

Estrogen receptor sensitivity was not associated with HIV status, but HIV-positive patients were less likely than others to have hormone-sensitive disease, perhaps because they were younger than the other patients. No statistically significant difference was detected in stage at presentation, histologic subtype, tumor grade, or nodal involvement between the HIV-positive group and the other groups. We found little support for the notion that HIV altered the breast cancer phenotype, in accordance with the phenotype described by Hurley et al^14^ and Sarhan et al.^22^

Our HIV-positive patients who received chemotherapy tolerated it well. Earlier case reports on HIV-positive patients receiving systemic anthracycline-based therapy described adverse outcomes, including neutropenic sepsis, acute respiratory distress syndrome, *Candida* esophagitis, and death.^14,23^ Our patients started ART at initiation of chemotherapy regardless of CD4 count, per national ART guidelines. We believe that with ART, most HIV-positive patients who are otherwise eligible for chemotherapy can benefit from it.

Although the literature implies that grade 3 or 4 myelosuppression is common among HIV-positive patients with breast cancer receiving chemotherapy, only 26.4% of our HIV-positive patients on chemotherapy (but none of the HIV-negative patients on chemotherapy) experienced grade 3 or 4 lymphopenia. They did not require dose adjustments, and they completed treatment. No dose-limiting toxicity was observed in the neutrophil count, and only two patients developed grade 3 neutropenia. A single case of grade 4 toxicity was observed in the hemoglobin level of a patient on a nevirapine-based ART regimen.

Failure to return to the hospital for treatment is not uncommon in South Africa. Many patients who come to Tygerberg Hospital are members of the Xhosa tribe residing in the rural Eastern Cape. Many of these patients speak only Xhosa, do not have access to telephones, have never seen the inside of a hospital, and travel more than 800 km to receive care at Tygerberg Hospital, which is the closest public hospital with full oncology services. Few patients have the means to return even once, let alone every 21 days, to receive chemotherapy. In our cohort, patients with and without HIV seemed to be equally affected by socioeconomic barriers to care. Further research is needed to help us understand and address the barriers faced by our patients.

Our study was not designed to determine whether HIV either prevented or promoted the development of breast cancer. All we observed in our HIV-positive patients with breast cancer was the coexistence of two chronic diseases. To date, the only association between CD4 count and the relative risk of developing a malignancy has been shown in cancers with a known viral pathogenesis, the so-called AIDS-defining cancers.

Among our HIV-positive patients who received chemotherapy, the CD4 count declined by a third of pretreatment values, from a median of 477 cells/µL (range, 234 to 807 cells/µL) to a median nadir of 333 cells/µL (range, 62 to 713 cells/µL). Lymphoma studies published in the pre-ART era established the perception that HIV-positive patients who received chemotherapy were at high risk for persistent, severe compromise of immunologic function and poor outcomes.^24^ In AIDS Malignancies Consortium Trial 010, an excess risk of death was associated with postchemotherapy opportunistic infections in patients with CD4 counts less than 50 cells/µL.^25^ However, in more recent studies, despite initial low CD4 counts, patients receiving ART tolerated chemotherapy without significant toxicity.^26-28^ Further studies are needed to confirm these findings among patients with non–HIV-related cancers. South Africa is an ideal setting in which to conduct such studies because of its disproportionate burden of HIV.

The greatest limitation of this study was that it was based on chart reviews. The chart data were not collected for the purpose of evaluating the effects of HIV on breast cancer phenotypes and outcomes. In addition, because CD4 values were not determined at set intervals after completion of treatment, we have no data on the longer term effects of systemic chemotherapy on the progression of HIV and on patient survival.

Among the patients in our sample, those who were HIV positive were diagnosed at a younger age than other patients with breast cancer and were predominantly of African ethnicity. The age distribution of HIV-positive patients with breast cancer seems to correspond to the age distribution of HIV-positive women in the general population. Although HIV-positive patients were more likely than others to have PR-negative cancers, that difference in phenotype was likely to have been a result of age and ethnicity rather than HIV itself. The HIV-positive patients who received systemic chemotherapy tolerated it well, experienced no significant hematologic toxicity, and developed no opportunistic infections during treatment. The mean decline in CD4 cell count during chemotherapy was 30%. Our findings should reassure patients with HIV and breast cancer, as well as their providers, that, with ART and careful monitoring, they can benefit from appropriate chemotherapy.
